# Fractional Dynamics Identification via Intelligent Unpacking of the Sample Autocovariance Function by Neural Networks

**DOI:** 10.3390/e22111322

**Published:** 2020-11-20

**Authors:** Dawid Szarek, Grzegorz Sikora, Michał Balcerek, Ireneusz Jabłoński, Agnieszka Wyłomańska

**Affiliations:** 1 Faculty of Pure and Applied Mathematics, Hugo Steinhaus Center, Wroclaw University of Science and Technology, Wybrzeże Wyspiańskiego 27, 50-370 Wroclaw, Poland; 234856@student.pwr.edu.pl (D.S.); grzegorz.sikora@pwr.edu.pl (G.S.); michal.balcerek@pwr.edu.pl (M.B.); 2 Department of Electronics, Wroclaw University of Science and Technology, B. Prusa 53/55, 50-317 Wroclaw, Poland; ireneusz.jablonski@pwr.edu.pl

**Keywords:** anomalous diffusion, fractional Brownian motion, estimation, autocovariance function, neural network, Monte Carlo simulations

## Abstract

Many single-particle tracking data related to the motion in crowded environments exhibit anomalous diffusion behavior. This phenomenon can be described by different theoretical models. In this paper, fractional Brownian motion (FBM) was examined as the exemplary Gaussian process with fractional dynamics. The autocovariance function (ACVF) is a function that determines completely the Gaussian process. In the case of experimental data with anomalous dynamics, the main problem is first to recognize the type of anomaly and then to reconstruct properly the physical rules governing such a phenomenon. The challenge is to identify the process from short trajectory inputs. Various approaches to address this problem can be found in the literature, e.g., theoretical properties of the sample ACVF for a given process. This method is effective; however, it does not utilize all of the information contained in the sample ACVF for a given trajectory, i.e., only values of statistics for selected lags are used for identification. An evolution of this approach is proposed in this paper, where the process is determined based on the knowledge extracted from the ACVF. The designed method is intuitive and it uses information directly available in a new fashion. Moreover, the knowledge retrieval from the sample ACVF vector is enhanced with a learning-based scheme operating on the most informative subset of available lags, which is proven to be an effective encoder of the properties inherited in complex data. Finally, the robustness of the proposed algorithm for FBM is demonstrated with the use of Monte Carlo simulations.

## 1. Introduction

Many single-particle tracking data related to the motion in crowded environments exhibit anomalous diffusion behavior [1,2]. This behavior is also visible in various phenomena such as finance [3,4], ecology [5], hydrology [6], and biology [7], as well as meteorology and geophysics [8,9]. Anomalous diffusion behavior is manifested by deviations from the laws of Brownian motion (BM). One of the most common definitions of the anomalous diffusion process is expressed in the nonlinear behavior of its second moment:

(1) $$\begin{array}{c} E\left[X^{2}\left(t\right)\right]∼t^{\alpha }, \end{array}$$

where α is the so-called anomalous diffusion exponent. When α=1, the process is classified as diffusion, while for α≠1, we call it anomalous diffusion. More precisely, for α<1, it is called subdiffusion, while for α>1, it is superdiffusion. It should be mentioned that anomalous diffusion can be also related to the non-Gaussian probability density function of the corresponding process; for instance, see [10,11,12,13].

The class of anomalous diffusion processes is very rich. The most classical anomalous diffusion models are fractional Brownian motion (FBM) [8,14], Lévy stable motion [15], continuous-time random walk [16,17], and the subordinated processes (also called time-changed processes) [18,19,20,21,22,23,24,25]. We also mention here the processes with time- or position-dependent diffusion coefficients such as scaled Brownian motion [26,27] or heterogeneous diffusion models [28], as well as the superstatistical process [29] or diffusing diffusivity models (also called Brownian yet non-Gaussian diffusion process) [30]. We also refer the readers to the articles [31,32,33,34] and the references therein.

In the case of experimental data with anomalous dynamics, the main problem is first to recognize the type of anomaly and then to reconstruct properly the physical rules governing such a phenomenon. The main challenge is to identify the process from short trajectory inputs. Various approaches to address this problem can be found in the literature; for instance, see [35,36,37,38,39,40,41,42]. One of the simplest and most efficient approaches is based on the theoretical properties of the sample autocovariance function (ACVF) [43]. It is known that the ACVF is the characteristic that determines completely the centered Gaussian process. Thus, its sample version is a proper tool for the testing and estimation of the parameters of this process. The approach presented in the literature [43] is effective; however, it does not utilize all of the information contained in the sample ACVF for a given trajectory, i.e., only values of statistics for selected lags are utilized. Therefore, an evolution of this approach is proposed in this paper.

Herein, we compared three approaches that apply the ACVF for estimation of the anomalous diffusion exponent. The first one, the so-called *naive* method, uses the ACVF in one specific lag to estimate the anomalous diffusion exponent of the given process. The second algorithm is based on the ACVF corresponding to the vector of the selected lags. The last technique is based on the sample ACVF information extracted with the most informative subset of available lags. The designed novel method in the third approach is intuitive and it uses information directly available in a new fashion. Information retrieval from the sample ACVF vector is performed here with a learning-based scheme operating on the most informative lags, i.e., a feedforward neural network (FNN) [44] is designed and applied for solving the regression task. This approach has been proven to be an effective encoder of the properties inherited in complex data [45,46,47]. The goal is to preliminarily assess (using computer simulations) the predictive properties of an FNN for the estimation of the anomalous diffusion exponent based on a short data set. This exercise provides evidence of the performance of the simple version of the neural network (NN) adapted for the defined regimes (anomalous diffusion) and the ACVF vector, which is a projection of a valid complex and real trajectory, e.g., for particle movement in a solution. The reported results can be further enhanced by the exploitation of adaptive mechanisms inserted into recurrent neural networks (RNNs) and/or the detailed and proper inferences of multiscale pattern(s) for deep learning [1,2,3,4,5]. The advantage of the application of an FNN to the task defined above is that a trained neural network model can be a robust and efficient estimator for anomalous diffusion exponents, e.g., complex relations hidden in ACVF data can be extracted within one-step, concluding in a trained feedforward neural network.

The robustness of the introduced algorithm based on the ACVF and NN methods in comparison to the known ACVF-based techniques is demonstrated herein for the exemplary Gaussian process using Monte Carlo simulations. We considered the FBM as an exemplary model with fractional dynamics; that is, the Gaussian process with stationary increments and the so-called self-similar property parametrized by the Hurst exponent H=0.5α, where α is the anomalous diffusion exponent given in (1).

The main goal of the paper was to prove that the incorporation of intelligent-based algorithms into classical estimation schemes can shed new light on the investigation of the anomalous diffusion phenomenon. Moreover, the classical tools enhanced by artificial intelligence (AI) methods are more effective in comparison to the known statistical algorithms used for anomalous diffusion parametrization.

The rest of the paper is organized as follows: In Section 2, we outline the definition of the fractional Brownian motion and the exemplary Gaussian process with anomalous diffusion behavior. Next, in Section 3, we discuss two of the estimation methods for the Hurst exponent based on the ACVF that are commonly used in various applications. In the next section, we present a new approach for the estimation of the *H* index. Namely, in the new algorithm, we combined the ACVF and NN methods. To demonstrate the effectiveness of the new approach, in Section 5, we present a simulation study where we compare the three considered algorithms for the estimation of the Hurst exponent. The last section concludes the paper and presents a future study.

## 2. Fractional Brownian Motion

Fractional Brownian motion (FBM) $\left\{X_{H}\left(t\right),\;t\geq 0\right\}$ with the Hurst index H∈(0,1) is a continuous and centered Gaussian process defined through the following Langevin equation [14,48,49,50]:

(2) $$\begin{array}{c} \frac{dX_{H}\left(t\right)}{dt}=D\xi_{H}\left(t\right), \end{array}$$

where the parameter *D* is the diffusion coefficient. In Equation (2), $\left\{\xi_{H}\left(t\right),\;t\geq 0\right\}$ is the fractional Gaussian noise process with the autocorrelation function satisfying the following:

(3) $$\begin{array}{c} \mathrm{Corr}\left(\xi_{H}\left(0\right),\xi_{H}\left(t\right)\right)∼2H\left(2H-1\right)Dt^{2(H-1)},\;t\geq 0. \end{array}$$

The FBM was introduced by Kolmogorov in 1940 (see [8]). FBM is the only Gaussian process with the self-similar property. Because the FBM is a centered Gaussian process, it can be also defined through the ACVF that, in this case, is given by [14]:

(4) $$E\left[X_{H}\left(t\right)X_{H}\left(s\right)\right]=\frac{1}{2}D\left(t^{2H}+s^{2H}-{\left|t-s\right|}^{2H}\right),\;\mathrm{where}\;t,s\geq 0.$$

Thus, for the given t≥0, $X_{H}\left(t\right)∼N\left(0,Dt^{2H}\right)$. The FBM has stationary increments. Moreover, if H>1/2, then the increments of the process are positively correlated, while for H<1/2, they are negatively correlated. Moreover, for H>1/2, the FBM exhibits the so-called long range dependence, which means that the following property is satisfied:

(5) $$\begin{array}{c} \sum_{n=1}^{\infty }E\left[X_{H}\left(1\right)\left(X_{H}\left(n+1\right)-X_{H}\left(n\right)\right)\right]=\infty . \end{array}$$

**Figure 1:**
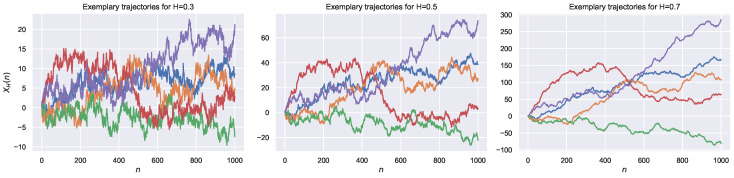
Exemplary trajectories for H∈{0.3,0.5,0.7}.

As can be seen, for H=1/2, the FBM reduces to the ordinary Brownian motion (BM). It should be mentioned that the FBM is considered one of the classical processes used to describe the anomalous diffusion phenomenon. Indeed, for H<1/2, it exhibits subdiffusion behavior, while for H>1/2, it shows superdiffusion behavior. To see the differences between the behavior of the trajectories corresponding to different anomalous types, in Figure 1, we demonstrate the exemplary trajectories of FBM for H=0.3 (subdiffusion), H=0.5 (diffusion), and H=0.7 (superdiffusion).

## 3. ACVF-Based Methods for the Estimation of the Hurst Exponent

In this article, we consider three approaches that utilize the ACVF in the estimation of the Hurst parameter *H*. Here, we depict two approaches known from the literature. The last technique is described in detail in the next section.

The methods presented in this section are based on the sample version of the ACVF. Let us consider the trajectory of FBM, $X_{H}=\left\{X_{H}\left(1\right),X_{H}\left(2\right),\cdots ,X_{H}\left(n\right)\right\}$, and the corresponding sample of increments, $\xi_{H}=\left\{\xi_{H}\left(1\right),\xi_{H}\left(2\right),\cdots ,\xi_{H}\left(n\right)\right\}$, where $\xi_{H}\left(t\right)=X_{H}\left(t\right)\;-\;X_{H}\left(t-1\right)$ for t=1,2,…,n. The sample ACVF for $\xi_{H}$ is given by [51]:

(6) $$\begin{array}{c} {\hat{\gamma }}_{\xi }\left(\tau \right)=\frac{1}{n}\sum_{t=1}^{n-\tau }\xi_{H}\left(t\right)\xi_{H}\left(t+\tau \right),\;\tau =0,1,\ldots ,n-1. \end{array}$$

The statistic ${\hat{\gamma }}_{\xi }\left(\tau \right)$ is a rescaled estimator of the theoretical autocovariance function for $\gamma_{\xi }\left(\tau \right)$, corresponding to lag τ, where $\gamma_{\xi }\left(\tau \right)=E\left(\xi_{H}\left(1\right)\xi_{H}\left(1+\tau \right)\right)$. One can easily show that the statistic (6) is a biased estimator of $\gamma_{\xi }\left(\tau \right)$, namely:

$$\begin{array}{cc} E\left[{\hat{\gamma }}_{\xi }\left(\tau \right)\right] & =\frac{1}{n}E\left[\sum_{t=1}^{n-\tau }\xi_{H}\left(t\right)\xi_{H}\left(t+\tau \right)\right]=\\ \; & =\frac{n-\tau }{n}E\left[\xi_{H}\left(1\right)\xi_{H}\left(1+\tau \right)\right]=\frac{D(n-\tau )}{2n}\left({\left|\tau +1\right|}^{2H}{+|\tau -1|}^{2H}-2{\left|\tau \right|}^{2H}\right)=\frac{n-\tau }{n}\gamma_{\xi }\left(\tau \right). \end{array}$$

However, in our considerations, we used the biased version of the $\gamma_{\xi }\left(\tau \right)$ estimator due to its lower variance in comparison to the unbiased one.

In the literature, a few possibilities of estimating the Hurst parameter *H* using the ACVF have been presented. In the simplest approach, we considered only the first lag τ=1, and compared the statistic ${\hat{\gamma }}_{\xi }\left(1\right)$ with the desirable theoretical value of $\gamma_{\xi }\left(1\right)=\frac{D}{2}\left(2^{2H}-2\right)$. Thus, using the following relation:

$$\begin{array}{c} {\hat{\gamma }}_{\xi }\left(1\right)=\frac{D}{2}\left(2^{2H}-2\right), \end{array}$$

one can obtain the simple estimator of *H*:

(7) $$\begin{array}{c} \hat{H}=\frac{1}{2}\log_{2}\left({\hat{\gamma }}_{\xi }\left(1\right)\frac{2}{D}+2\right). \end{array}$$

As such an approach is simple, we refer to it as *naive* (the M1 method) in the following analyses. In this approach, the diffusion coefficient *D* is assumed to be known. However, in real applications, the *D* parameter can also be estimated as a sample variance of the vector ξ. As one can see, the ACVF for lag τ=1 includes the necessary information about the FBM process. Unfortunately, if the corresponding measurements are burdened by an additive error (i.e., some measurement noise), γ(1) changes accordingly, resulting in the need to consider more advanced techniques for the estimation of the *H* parameter; for instance, see [52,53] and the discussion therein.

In an alternative approach for estimating the *H* parameter, one can simultaneously use more lags τ. Thus, we can fit the function $\tau \mapsto \frac{D}{2}\left({\left|\tau +1\right|}^{2H}{+|\tau -1|}^{2H}-2{\left|\tau \right|}^{2H}\right)$ to the empirical ACVF ${\hat{\gamma }}_{\xi }\left(\tau \right)$ for the corresponding lags $\tau =1,2,\ldots ,\tau_{max}$ in the least squares sense, i.e., the estimator is calculated as follows:

(8) $$\begin{array}{c} \hat{H}={\arg\;\min}_{H\in (0,1)}\sum_{\tau =1}^{\tau_{max}}{\left[{\hat{\gamma }}_{\xi }\left(\tau \right)-\frac{D}{2}\left({\left|\tau +1\right|}^{2H}{+|\tau -1|}^{2H}-2{\left|\tau \right|}^{2H}\right)\right]}^{2}, \end{array}$$

for some maximum lag $\tau_{max}$. Again, if the diffusion coefficient is unknown, we can estimate it by considering argmin over (H,D)∈(0,1)×(0,∞). This approach is unfortunately much more complex, as it requires nonlinear regression methods. In the further analysis, we refer to this approach as the M2 method.

## 4. ACVF and NN-Based Methods for the Estimation of the Hurst Exponent

As mentioned above, three methods for the estimation of the Hurst exponent *H* are compared in this article. All of the identification algorithms are based on the ACVF. The first (M1) and the second (M2) methods were described in the previous section. In this section, we present the algorithm based on the ACVF and NN methods, denoted as the M3 method in the simulation study.

One might expect that the two proposed methods provide the best estimation results when the data follows the “pure” theoretical model, i.e., FBM. In reality, this is rarely the case—often, the observed trajectories are biased by a measurement error and/or various interleaving processes cover the anomalous diffusion component in the acquired signal. A purely statistical approach, such as in the M1 and M2 methods, can bring about limitations in real-world data applications, whereas artificial intelligence has shown its potential to overcome parasitic conditions in data from numerous fields of applications [45,54,55,56]. This triggers applications of learning-based schemes that enable weakening of the initial assumptions related to data properties and model building, providing good estimators for a wide range of anomalous diffusion regimes (i.e., for a wide range of *H* values). The assumed architecture for the neural network model and the training process are crucial aspects for efficient data exploration in artificial intelligent schemes, with the latter being of particular importance for NN model performance [44]. As a consequence, the input data properties used for the NN model training condition the reliability of the observed outputs—i.e., the value of the estimated *H* and its uncertainty in the reported study.

In the previous section, we assumed that the relationship between the data and the estimated parameter is known and given by Equations (7) and (8) for the M1 and M2 methods, respectively. Now, we propose to use a feedforward neural network as the predictor of this theoretical relationship, i.e., $E[H|\left\{{\hat{\gamma }}_{\xi }\left(\tau \right)\right\}]$. To be more precise, the FNN is proposed as the model of the hidden relationships in the experimental data. The last one means that obtaining a formal expression for the rules governing the phenomena in a real-world system is not the main subject of interest here, but encoded in the FNN model, these rules are used to enhance the reliability of the Hurst exponent estimation from the data that correspond to the FBM, according to our assumption. It is worth noting that the modeling of $E[H|X_{H}]$ or $E[H|\xi_{H}]$ is also possible with the NN-based approach; however, more sophisticated NN topologies are required to reconstruct the long dependency valid for the FBM model (for H≫0.5). Thus, dealing with long and varying input vector lengths is required to realize this task; models based on RNNs, long short-term memory (LSTM) neural networks [57], or other forms of intelligent recurrence should be used, which implies higher requirements regarding their training [58].

The information about the underlying process is concentrated in the first couple of lags of the sample ACVF. In this paper, 32 lags (including the 0th lag) were used as the input for the Hurst exponent estimation. In the next section, it is shown that this amount was sufficient for our study.

The proposed architecture of the neural network model consists of three hidden layers of consecutive sizes of 64, 64, and 32. The Swish-1 [59] activation function, defined as:

(9) $$\begin{array}{c} \mathrm{Swish}-\beta \left(x\right):=x\times \sigma \left(\beta x\right)=\frac{x}{1+e^{-\beta x}}, \end{array}$$

was used for the neurons in each layer—in many applications, this expression outperforms the other activation functions [59].

In the designed FNN predictor, the size of the first layer was conditioned by the number of lags corresponding to the ACVF used during the experiment (i.e., 32 neurons were inserted into the first layer) and the output layer consisted of one neuron, which produced the *H* estimator as the model response. Since the estimated value of the Hurst exponent was within the range of H∈(0,1) and the FNN model designed for its estimation can produce all real numbers as the output, post-processing transformation needed to be applied to the response of the output neuron. Here, the sigmoid function, defined as:

(10) $$\begin{array}{c} \sigma \left(x\right):=\frac{1}{1+e^{-x}}, \end{array}$$

was used. The function given in (10) projects all real numbers to the interval (0,1).

To boost the FNN training process, the Adam optimization algorithm [60] was applied as it quickly converges to a minimum [61,62,63,64]. The mean squared error (MSE) was used to quantify the prediction error of the FNN model.

## 5. Simulation Study

The efficiency of the three methods (M1, M2, and M3) designed for *H* estimation is demonstrated in this section using computer simulations. Cholesky decomposition [65] was used for the generation of the FBM trajectories, since it allows to simulate outputs with extreme *H* parameter values (unlike Davies–Harte [66], which can fail to generate small samples for *H* parameters close to 1). Regarding the practical usefulness of the designed procedures, their efficiency can be expressed in the context of the length of the input trajectory required to estimate *H* with expected reliability. This study was performed for trajectories of various lengths, and the results are reported below.

The two statistical methods described in Section 3 were ready to use, whereas the designed feedforward neural network needed to be trained in advance, for which training and validation datasets were prepared.

The training dataset was formed with 1,572,64 FBM trajectories generated during computer simulations. This set consisted of vectors of different lengths (from 32 up to 1024) and referred to different *H* parameter values. For every trajectory length, N∈{32,64,128,256,512,1024}, 262,144 trajectories were generated using computer simulations (262,144 × 6 = 1,572,864), each with the *H* parameter selected randomly from the uniform distribution U(0,1). Next, for each trajectory $\xi_{H}$, the ACVF (biased estimator (6), as introduced earlier) was calculated, resulting in a set of ACVFs of lengths *N* (*N* lags—τ∈{0,1,…,N−1}) with corresponding *H* parameters. Using the same procedure, the validation and test subsets were generated, each of a size of 196,608 (32,768 × 6 = 196,608).

**Figure 2:**
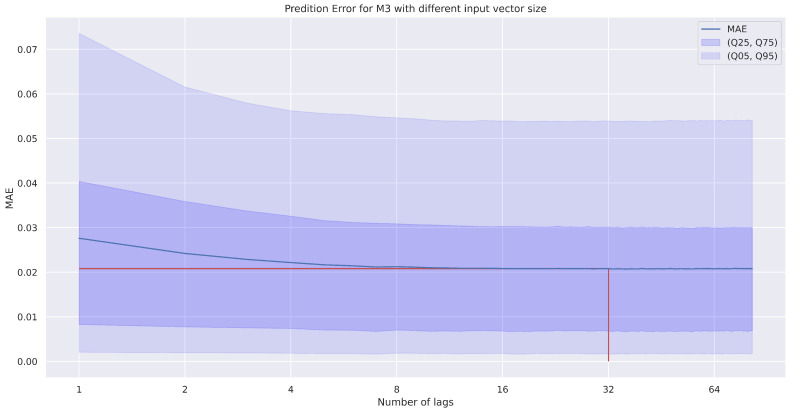
The MAE calculated for the M3 method when a different number of lags is used as the feedforward neural network (FNN) input (determining the size of the input layer of the FNN), depending also on the selected quantile; the number of lags (32) selected for use in the paper are marked with a red line.

The length of the ACVF vectors for the NN training was limited to 32 first lags (namely, τ∈{0,1,…,31}). This selection was preceded by the calculation of MAE prediction error, such as in Figure 2—more input samples do not decrease the error, whereas a smaller input size increases the MAE value.

To train the FNN (the architecture of the FNN as described in Section 4), input data were gathered into batches of 64 (the number of training examples used to calculate weight updates). The total number of NN parameters was 8385, trained for 13 epochs (the number of times that the training algorithm operated on the entire training dataset), for a total of 93s×13epochs≈20min. The number of epochs was selected dynamically using the early stopping method [67]. Since the model did not improve the prediction error significantly after the third epoch, the training procedure could be stopped then (then, the training time would be 5 min). Calculations were performed on a PC with Intel Core i7 (3.7 GHz, 6 cores, 12 threads) and RAM of 64 GB.

The test dataset was used to compare the M1, M2, and M3 methods—for M1, the first lag was used (τ=1); for M2, the set of lags was $\tau \in \{0,1,\ldots ,\tau_{max}\}$ (if not stated differently, $\tau_{max}=31$); M3 always used 32 lags (τ∈{0,1,…,31}). The metrics used were the absolute error and (for the aggregated results) the mean absolute error.

**Figure 3:**
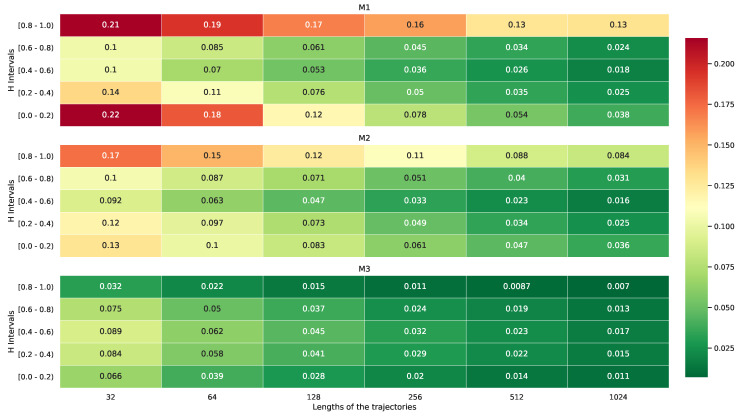
MAE heatmap for the M1, M2, and M3 methods depending on the length of the input trajectory and the value of the Hurst exponent applied during the computer simulations.

Figure 3 provides a comparison of the MAEs for the three methods (i.e., M1, M2, and M3) when dealing with trajectories of different lengths and with the diffusion characteristic (*H* parameter), grouped by the true parameter *H* into the bins [0–0.2), [0.2–0.4), [0.4–0.6), [0.6–0.8), and [0.8–1).

The conclusion is that the NN-based approach (M3) is more efficient than the other schemes studied in the paper, as it did not need long trajectories to estimate the Hurst exponent *H* with a minimized/minor error. For *H* close to 0 or 1, there was a significant difference in the MAEs between the considered methods—although the NN approach achieved a similar level of error to the other methods when fed samples of a size of 64 for the estimation, M1 and M2 struggled to deliver equivalent performance for longer inputs, i.e., up to 16-times longer trajectories, as was shown during the computer simulations. It is worth noting that the estimation error for the Hurst exponent in the diffusion case (i.e., H≈0.5) was similar for all of the considered approaches. In summary, since M1 estimated *H* reliably when using only the first lag, which was also true for the other methods, it was possible to distinguish between normal and anomalous diffusion using the information contained in the first lag or the first few lags.

**Figure 4:**
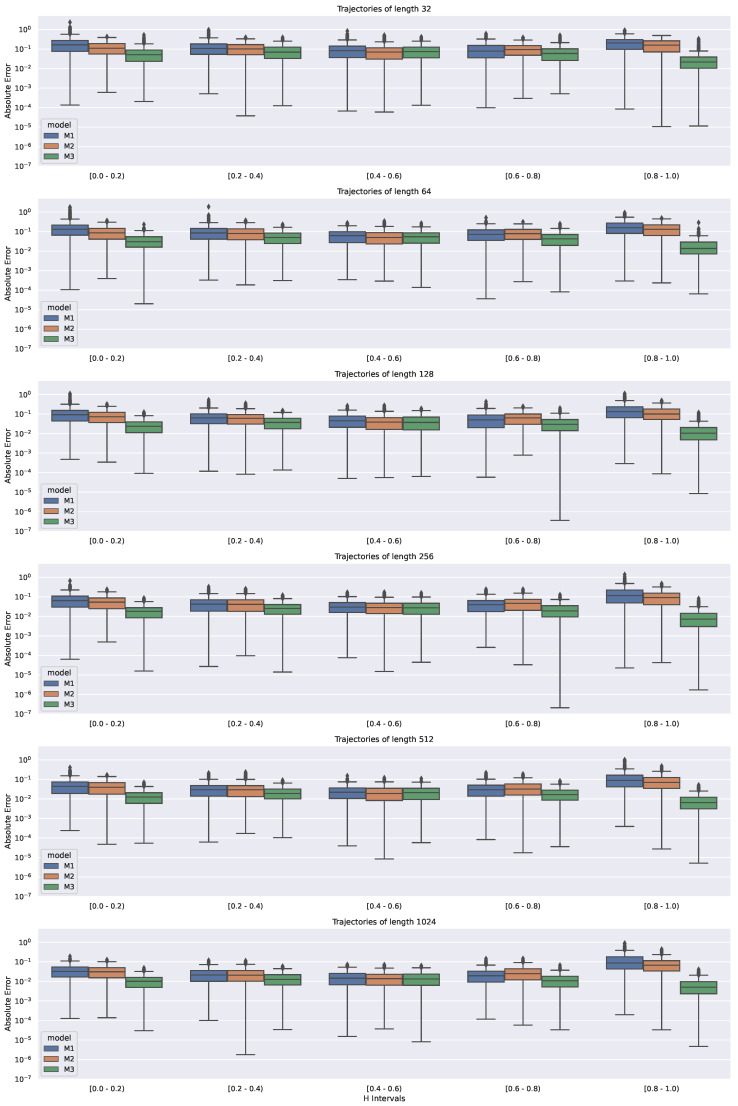
Absolute error calculated for the M1, M2, and M3 methods when fed with simulated input trajectories of different lengths and representing various modes of anomalous diffusion regimes (encoded with the *H* value).

To further compare these methods, the distribution of the (absolute) prediction error is shown in Figure 4. A logarithmic scale was applied to the y-axis in the boxplots to distinguish the performance of the following algorithms. The figure is divided into six parts, each reporting on the performance of the M1, M2, and M3 methods. In this way, it was possible to analyze how the distributions of the prediction error varied for each of these methods, the different lengths of the input trajectories, and also the different diffusion types (i.e., superdiffusion and subdiffusion); similarly to Figure 3, the input data were grouped evenly into five bins in reference to the true value of the *H* parameter.

Figure 4 depicts the spread of the estimation error and also reports on the number of outliers. The obtained results prove that the NN-based algorithm can more reliably estimate the *H* value for its following ranges (thus regimes of anomalous diffusion behavior) than in the two classical (M1 and M2) methods.

When it comes to the analysis of the multidimensional spread of the observed distributions, M3 performed similarly to the other methods. However, it is worth noting that M1 could not be used to obtain reliable results for some anomalous diffusion regimes, i.e., in several cases in Figure 4, the prediction errors were over 0.5 (which means that M1 could not distinguish between sub- and superdiffusion in these cases). There were also some cases in the presented study when all of the methods working with the smallest considered sample size (32) struggled to distinguish between subdiffusion and superdiffusion. Nonetheless, M3 applied to two-times longer trajectories (i.e., 64 samples inserted as the input) performed reliably and efficiently through the whole range of *H* values.

It would be advantageous here to understand what makes M3 outperform the M2 algorithm. The most straightforward explanation relies on the fact that the input information contributes to outputs in various ways in M2 and M3. Namely, although M2 and M3 use the same input information, M2 explores each lag with the same importance, which is not the case for the FNN. The neural network-based method can learn inhomogeneous relationships contained in historical data, focusing on a specific subset of lags and leaving others with less influence on the observed output. This is exactly how the intelligent unpacking mechanism works in the M3 method.

**Figure 5:**
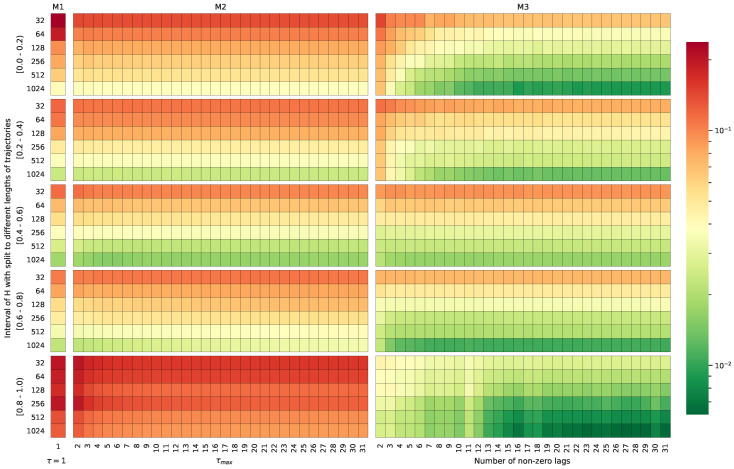
MAE heatmap calculated for the M1, M2, and M3 methods depending on the length of the input trajectory, $\tau_{max}$, and for the following ranges of Hurst exponent values.

This phenomenon can be clearly observed in Figure 5, where the MAE is compared for the methods working with trajectories of different lengths, for different *H* parameter values, and various numbers of lags used during the calculations. In the case of the M1 method, the results are presented only for τ=1, as there was no possibility to expand or shrink the set of lags here. M2 used lags up to $\tau_{max}$ for the Hurst exponent estimation, i.e., ($\tau \in \{0,1,\ldots ,\tau_{max}\}$) with $\tau_{max}\in \left\{2,3,\ldots ,31\right\}$. The number of used lags could not be reduced for the M3 method since it required exactly 32 lags. To overcome this problem, a selected number of the first values of the calculated lags for the ACVF (this number is later referred to as non-zero lags) were extracted. The remaining lags were reset to zero during the calculations. This means that the relationships valid for the raw data were cut at the level of some lags, resembling the diffusion case H=0.5 (i.e., there is no inter-dependency and the ACVF is equal to 0). All of these contributed to weakening the anomalous behavior (filling further lags with zero diminished/removed the long-range dependencies); thus, the reliability of the used methods for prolonged input data sequences could be improved, especially in the case of the M3 algorithm (see Figure 3 and Figure 4).

Figure 5 proves that using at least 20 lags was sufficient to minimize the error of *H* estimation in M3. However, the prediction error imperceptibly decreased for M2 with the addition of consecutive lags to the input. One of the explanations for this observation might be that the magnitude of the contribution to the observed outputs (here, the value of the Hurst exponent) was smaller for the higher-order lags than for the first few lags. This means that the feedforward neural network more efficiently unpacked the information about the anomalous diffusion process than the M1 and M2 statistical schemes.

## 6. Summary and Conclusions

Anomalous diffusion is a complex phenomenon observed in physical systems. This complexity is inherited in recorded data, which, in practice, can be additionally corrupted by measurement noise. Moreover, anomalous diffusion components can emerge from a bunch of other regimes manifested in the observed system (and thus, in recorded data). Unpacking and disentangling the information contained in such data is a challenge, especially because complex interrelations are typically encoded in a small number of data samples. The statistical modeling of anomalous dynamics is quite well established and is concerned with the approximation of nontrivial patterns by the anomalous diffusion exponent. The problem is that for complex systems and the limited a priori knowledge available, the statistical inference can bring about non-robust estimation, especially when noisy components occur in data.

In recent years, there has been increasing interest in the use of AI methods of data exploration in various areas of science and engineering, especially when dealing with complex systems and a limited amount of preliminary information. In this paper, we proposed the application of a simple feedforward neural network to the quantification of anomalous dynamics. Namely, we compared this AI-based approach with statistical modeling, which allowed us to conclude that a combination of these two data exploration methods can decrease the estimation error of the anomalous diffusion exponent.

Herein, we considered the FBM, as it is one of the classical Gaussian processes that can be used for the description of anomalous diffusion behavior. Moreover, the FBM for special cases reduces to the classical BM, and thus is also useful for the analysis of diffusion processes.

Our approach is based on the ACVF, which completely describes the zero-mean Gaussian process. Thus, we selected the sample version of this statistic as a base for the estimation methodology. Via a simulation study, we proved that the classical approaches that utilize the sample ACVF are effective; however, when the process under consideration becomes anomalous diffusion, then their efficiency decreases. Thus, there is a need to incorporate more advanced techniques. In this article, we addressed such problems and proposed a simple modification of the classical ACVF-based approaches through the inclusion of NN-based methodology. Our simulations showed that the introduced estimation method outperformed the other considered approaches, especially for short trajectory lengths. This message is crucial for practical applications, where the real trajectories may be relatively short. In this case, the method based on a combination of ACVF and NN techniques seems to be more effective in contrast to the classical algorithms. It should be highlighted that although the presented approach in this paper is based on the ACVF, this methodology can be applied to any other statistic that is crucial for the estimation of anomalous diffusion processes, e.g., mean squared displacement, p-variation, and ergodicity breaking parameter. Moreover, the potential applicability of the described approach is much wider than the anomalous diffusion field. It could bring new insight into studies of physical systems with various properties reflected and decoded in the corresponding statistical quantities, i.e., long-range dependence, ergodicity breaking, or self-similarity. Additionally, the FBM was considered here only as an exemplary anomalous diffusion process, and the presented approach can be used for any other models with anomalous diffusion behavior. The designed method can also also used not only for estimation purposes, but also for the classification of the anomalous diffusion model governing physical phenomena. It could be directly achieved by the NN learning various ACVF formulas that uniquely define different Gaussian processes. Finally, outside of the Gaussian world, other appropriate statistics that evidently and precisely detect proper non-Gaussian models can be effectively brought into the identification process. Thus, the introduced approach is universal in many areas and can be extended in various directions.

The combination of known statistical algorithms with the deep learning methodology is not a new idea in the area of anomalous diffusion phenomena analysis. Many authors have recognized that simple statistical methods in some cases seem to be inefficient for the proper identification or parametrization of the anomalous diffusion model, especially for recordings with short-length trajectories. In recent years, in the literature regarding anomalous diffusion processes, one can find the application of intelligent methods that enhance the classical approaches. In the physical sciences, deep learning methods have ound very interesting applications. We mention here the new approaches based on the artificial NN algorithms (i.e., [68,69,70,71,72,73]) or the general machine learning methods applied to fractional dynamics analysis (i.e., [74,75,76,77]). However, to the best of our knowledge, a combination of ACVF- and NN-based methods has not been presented in the context of anomalous diffusion analysis.

In a future study, we plan to analyze the influence of additive noise on the estimation results for a model disturbed by external force. The same problem has been considered previously (e.g., in [53]), where the effectiveness of the time-averaged mean square displacement-based approach for the *H* parameter estimation was analyzed for the FBM with additive noise. Another future study will be related to the combination of the other time-averaged statistics [78] and deep learning methodology for the estimation problem in the anomalous diffusion regime.
